# Thomas Lea Buckingham, M. D., D. D. S.

**Published:** 1883-10

**Authors:** 


THOMAS LEA BUCKINGHAM, M. D., D.D. S.

Died at his residence, No. 1228 Arch Street, Philadelphia, Tuesday, September 4th, 1883, Prof. T. L. Buckingham, in the 68th year of his age.

This announcement will fall with surprise upon the professional and personal friends of this venerable and beloved teacher and dentist. It was but so recently that he met his brethren at the meeting of the American Dental Association at Niagara, that it seems scarcely possible that we are to meet him no more on earth. At that meeting he took his usually active part in the discussions, and showed the same quickness of apprehension and sound judgment for which he has ever been distinguished, though his form seemed more bent than usual, and his step had lost the elasticity of earlier days. Soon after he returned home he began rapidly to fail, and on September 4th death closed the scene. The immediate cause of his death was failure of the vital powers through softening of the brain.

Dr. Buckingham was born in Delaware, March 9th, 1816. He obtained his education in a country school near Brandywine Springs, and spent his early life in mechanical pursuits, until about his twenty-seventh year, when he removed to Wilmington, Delaware, and began the study of dentistry in the office of Dr. A. C. Reynolds.

In 1845 he removed to Philadelphia, where he entered into partnership with Dr. Lee, and in 1846 commenced practice alone.

In 1850 he became a member of the Pennsylvania Association of Dental Surgeons, and was seldom thereafter absent from a meeting until his death.

In 1851 the Philadelphia College of Dental Surgery was organized, and Dr. Buckingham was assigned to the chair of Mechanical Dentistry. This college was merged into the Pennsylvania College of Dental Surgery in 1856, and he held the same chair, but in 1857 he exchanged for that of Chemistry, and in this position he remained until his death, being at that time the oldest continuous dental teacher in the United States, and probably the world.

He was one of the founders of the *Dental Times*, and was a liberal contributor to its columns. He graduated in medicine in 1851, and in 1853 received the degree of Doctor of Dental Surgery from the Baltimore Dental College. He was President of the American Dental Convention in 1860, and in 1874 of the American Dental Association.

We gladly give place to the following very just estimate of his character as a man, by one of our associates:

"Another excellent and faithful man has been called to the spirit land. Professor Buckingham was one of the widest known and most popular members of our fraternity. Few men have done more for the benefit of our calling than did this estimable pioneer of dental instruction.

"Future meetings of the American Dental Association will miss his genial and fatherly presence, and his many old friends will miss the hearty grasp of the proffered hand that betokened an affectionate welcome. He was ever a conspicuous figure in the various professional gatherings of his city and State, and frequently visited meetings of societies in other places.

"With the Pennsylvania College of Dentistry he was long identified, and so wedded was he to its interests that to think of one would suggest the other.

"Although at times exceedingly earnest in giving expression to his views, and decidedly impressive in his teachings, gentleness and unobtrusiveness were marked characteristics of his nature. So kindly in disposition and so considerate was he of the feelings of others that he made no enemies, while his friends were legion.

"'None knew thee but to love thee,
Nor named thee but to praise.'

"Blessed be the memory of so good a man." C. E. F.

